# Service Availability and Readiness Assessment of Facility-Based Management of Severe Acute Malnutrition in Children in a Public Healthcare Setting in Jorhat, Assam

**DOI:** 10.7759/cureus.88746

**Published:** 2025-07-25

**Authors:** Dimpymoni Saikia, Basanta B Das, Bishnu R Das, Mridusmita Nath

**Affiliations:** 1 Community Medicine, Jorhat Medical College, Jorhat, IND; 2 Pediatrics, Jorhat Medical College & Hospital, Jorhat, IND

**Keywords:** mixed method study, nutrition rehabilitation centre, service availability and readiness assessment, severe acute malnutrition, thematic analysis

## Abstract

Background: Wasting is one of the most concerning growth and development issues in India, as in other parts of the world. Such children require carefully calibrated nutrition, together with management of co-morbidities. Both community and facility-based management are available for addressing the problem. Nutrition rehabilitation centres (NRCs) have been set up at health facilities in many districts to provide interventional measures to those malnourished children who require facility-based care. However, effective management of a case at the facility level with limited resources is always a challenge.

Objectives: To assess the service availability and readiness of facility-based management of children with severe acute malnutrition (SAM) in an NRC of Jorhat, and to identify the opportunities and barriers to facility-based management of SAM children.

Materials and methods: A descriptive (exploratory) qualitative study was done in the NRC of Jorhat for a period of six months, including six key informants and 12 caregivers. The data were collected through key informant interviews (KIIs) and in-depth interviews (IDIs) using semi-structured KII and IDI guides, as well as an observation guide to collect data on facility readiness, which was done by both observation and interview methods. Thematic analysis was done through inductive coding.

Result: The facility had a deficiency of helper/attendant and medical social worker (MSW) and lacked resuscitation and suction equipment. No separate sleeping arrangements were present for the caregivers. Other than these, sufficient ward equipment, kitchen, and pharmacy supplies were present. The major opportunities observed were the support from the medical college administration and the district health administration. The strength was a functioning NRC with dedicated staff. The major challenges from the facility side were a shortage of supporting staff and a shortage of some emergency equipment. From the beneficiary side, the major challenges were long duration of stay, financial loss, and poor nutritional awareness.

Conclusion: The present study has highlighted various challenges, both from the healthcare provider and beneficiary side, involving diverse areas such as human resources, financial issues, essential supplies, workload, nutritional awareness, and time constraints. However, all the beneficiaries expressed satisfaction with the services in the NRC.

## Introduction

Wasting is a worldwide burden. In the year 2022, globally, 45 million children under five years were wasted, of which 13.7 million were severely wasted. This translates into a prevalence of 6.8% and 2.1%, respectively. More than half of all children affected by wasting lived in South Asia. At 14.8%, South Asia’s wasting prevalence represents a situation requiring a serious need for intervention with appropriate treatment programmes [1]. India as a whole is presently combating the double burden of malnutrition, with both undernutrition and increasing overnutrition. At present, in India, 32.1% children under five years of age are underweight, and 35.5% are stunted. The National Family Health Surveys (NFHS) over the years have shown that the prevalence of severely wasted (weight-for-height) children increased from 6.4% in NFHS-3 (2005-2006) to 7.5% in NFHS-4 (2015-2016) and 7.7% in NFHS-5 (2019-2021) [2]. While in Assam, the prevalence increased from 6.2% in 2015-2016 to 9.1% in 2019-2020 [3].

Wasting represents a recent failure to receive adequate nutrition, resulting in failure to gain weight or actual weight loss. Severe wasting or severe acute malnutrition (SAM) is defined by a weight for height Z score less than -3 of the WHO growth standards median or visible wasting or mid-upper arm circumference (MUAC) less than 11.5 cm or presence of nutritional oedema [4]. Children with primary acute malnutrition are common in developing countries because of inadequate food supply caused by social, economic, and environmental factors. Secondary acute malnutrition is usually due to an underlying disease, causing abnormal nutrient loss, increased energy expenditure, or decreased food intake [5]. Consequences of malnutrition are diverse, affecting function of every organ system, including muscle, cardio-respiratory, gastrointestinal, immunity and wound healing, and psychological effects [6], SAM can be a direct cause of child death, or it can act as an indirect cause by dramatically increasing the case fatality rate in children suffering from such common childhood illnesses as diarrhoea and pneumonia. Children who are severely wasted are nine times more likely to die than well-nourished children [7]. Median case fatality rate in children with SAM is approximately 23.5%, which may reach 50% in oedematous malnutrition, attributed to various factors, even its management.

About 10% of SAM children require hospitalisation, while others are managed by community-based intervention. These 10% children require nutrition and other therapeutic interventions and should preferably be treated in specialised units like nutrition rehabilitation centres (NRCs) with skilled manpower and adequate resources for nutrition rehabilitation [7]. Management of SAM according to the WHO guidelines reduced the case-fatality rate by 55% in hospital settings. Recent studies suggest that community-based interventions such as ready-to-use therapeutic foods can be used to manage SAM in community settings [8]. In 2005, the National Rural Health Mission (NRHM) started setting up NRCs at health facilities in many districts of India. NRC provides interventional measures to the malnourished children and also educates the caregivers, i.e., the parents and guardians of the children, about the knowledge they should have to take care of the malnourished child. Facility-based management includes setting up and managing a functional space where these children are cared for within the health facility premises [7].

However, effective management of a case in a healthcare facility with limited resources is always a challenge. There can be potential barriers at the infrastructure, manpower, resource, or beneficiary level, which can affect the readiness of a facility in imparting services to the beneficiaries. Service availability and readiness assessment of any health facility can generate evidence to support the planning and management of a health system. Moreover, identification of the barriers can ensure effective policy development in the future. The study was conducted with the objectives to assess the service availability and readiness of facility-based management of severe acute malnourished children and to identify the opportunities and barriers to facility-based management of such children.

## Materials and methods

Study design

A descriptive (exploratory) qualitative study was conducted to assess the availability of resources and infrastructure in the NRC and to identify the challenges and barriers faced in facility-based management of SAM cases.

Study area

The study was conducted in the NRC of Jorhat. NRC in Jorhat was operationalised on 26th April 2015, with 10 beds. The facility functions under the National Health Mission, Assam. In Jorhat district, it is attached to Jorhat Medical College & Hospital (JMCH) campus. It caters to the population of the district and the nearby areas.

Study participants

Key informant interviews (KIIs) were conducted among the currently working manpower of the NRC. Healthcare providers for the interviews included nurses and the nodal officer (medical faculty) of the NRC. From the social sector, key informants included nutritionist-cum-counsellor and a cook posted in the facility.

In-depth interviews (IDIs) were conducted among the caregivers (parents and guardians) of SAM children admitted to the NRC during the time of visits of our study period.

Study duration

The study duration was six months from 1st November 2024 to 30th April 2025.

Sample size and sampling technique

In the study, a total of six participants were included as key informants and 12 caregivers were included for IDIs. We included the currently working manpower of the NRC as key informants of the study. The number of caregivers included in the study was dependent on data saturation during data collection as based on informational redundancy. All the study participants were adults.

Inclusion criteria

The inclusion criteria were as follows: (1) all the currently working manpower of the NRC as key informants; (2) all the caregivers of children or wards who were admitted for at least seven days in the facility.

Exclusion criteria

The exclusion criteria were as follows: (1) any key informant or caregiver who did not give consent; (2) any staff member of the NRC who was on leave during our study duration.

Data saturation

In the study, the saturation goal was based on category saturation. Data analysis was done simultaneously with data collection. Stopping criterion was set beforehand, and we stopped the data collection when three consecutive interviews could not produce any new category. This was adapted from the report presented by Francis et al. [9].

Developing, testing, and validating questionnaires

The IDI guides for key informants and caregivers were developed based on the steps mentioned in the document "Conducting Key Informant Interviews in Developing Countries" by Kumar [10]. The semi-structured KII guide and caregiver IDI guide were then validated by a senior member of our research team and a subject expert from the Department of Community Medicine, Jorhat Medical College. For the IDI guide, the content validity was done by translating it into the vernacular language and then retranslating it back to English by a bilingual expert. A pre-testing of the questionnaires was conducted by two members of the team using the questionnaires on a set of respondents to check inter-rater reliability.

The observation guide was developed based on the suggested standard requirements, as mentioned in the Operational Guideline on Facility-Based Management of Children With Severe Acute Malnutrition, Govt. of India [7].

Components of the KII Guide

Key informants’ views on the current working status of the facility, barriers and challenges faced by them during service delivery, any initiatives taken, their opinion on the same, and suggestions to remove the barriers.

Components of the IDI Guide

Caregivers’ views on their experience in facility-based management of SAM, as well as the challenges faced during the treatment of their children in the facility.

Components of the Observation Guide

Availability of manpower, required documents related to SAM, essential ward equipment, kitchen supplies, pharmacy supplies, and infrastructure and equipment required at the NRC.

Service availability and readiness assessment (SARA)

It is a standardised measurement tool developed by the WHO to assess and monitor the availability and readiness of healthcare facilities to deliver essential services. It provides a comprehensive evaluation of a health system's capacity to provide quality care by examining factors like infrastructure, equipment, human resources, and overall preparedness. In our study, the domains used to assess the service availability and readiness were based on the WHO tool [11]. The domains were bed capacity, manpower and their training, availability of required documents related to SAM, essential ward equipment required for NRC, kitchen supplies, pharmacy supplies, infrastructure and equipment required at the NRC, etc.

Occupation-related terminologies used in the study

Elementary Worker

Person involved in simple, routine, and often manual tasks that typically require limited personal initiative and judgment, like domestic helpers, labourers, street vendors, etc. [12].

Semi-skilled Worker

Person involved in an occupation in which their performance requires the application of skills gained by the experience on the job, which is capable of being applied under the supervision or guidance of a skilled employee, like drivers, plumbers, etc. [12].

Data collection method

Prior permission was taken from the head of the paediatrics department to carry out the survey. For assessment of the opportunities and barriers, the data were collected from health professionals and staff of the NRC and parents/guardians/caretakers by interview method with the help of the pre-mentioned semi-structured interview and observation guides after taking verbal consent of the interviewees. Confidentiality of the interviews was maintained. The interviews were held on a mutually decided specific date and time. An audio recording of the interviews was made. Any key informant who was on long leave was excluded from the study. An IDI was conducted among the caregivers of SAM children who were admitted to the facility for at least seven days. To ensure data validity and reliability, data triangulation was done by including interviews, observation, and cross-examination of the response by different respondents. The data collection from the caregivers was stopped when category saturation was reached.

Data analysis

For qualitative analysis, first, the data were transcribed manually from the audio recordings, and then thematic analysis was done through inductive coding. Coding, categories, and themes were first created by one researcher and then validated by a second researcher of the team.

Ethical clearance

Ethical clearance was obtained from the Institutional Ethics Committee (Human), Jorhat Medical College and Hospital, Jorhat. The ethical clearance number is EC/NEW/INST/2024/AS/0552, dated 13/09/2024.

## Results

Socio-demographic profile of the participants

There were six key informants and 12 caregivers in the study, among whom interview was conducted to understand the opportunities/strengths and barriers/challenges in the management of SAM children in a facility. Five out of the six key informants were between 31 and 40 years of age, and all except one were female. The majority of the key informants had been associated with facility-based management of SAM for over nine years. All the manpower working in the facility had received training regarding facility-based management of SAM, as shown in Table 1.

**Table 1:** Summary of the socio-demographic profile of key informants. ANM: auxiliary nurse midwife; GNM: general nursing and midwifery; APSC: Assam Public Service Commission; NHM: National Health Mission; SAM: severe acute malnutrition; NRC: nutrition rehabilitation centre.

Variables		Key informants
Age	20-30 years	1
	30-40 years	5
Sex	Male	1
	Female	5
Education qualification	Class 8^th^ passed (middle school)	1
	Diploma (ANM/GNM)	2
	Graduate	2
	Postgraduate (professional)	1
Designation	Assistant professor (nodal officer)	1
	Nutritionist cum counsellor	1
	Staff nurse	3
	Cook	1
Affiliation	APSC (medical faculty)	1
	NHM	5
Training on facility-based care of SAM	Received	6
Duration of association with the NRC	5-10 years	6
Duration of service in the current facility	<5 years	5
	5-10 years	1

Out of the 12 caregivers, eight were female. Most of the respondents were between 20 and 30 years of age, illiterate (50%), and engaged in elementary work (50%), as depicted in Table 2.

**Table 2:** Summary of the socio-demographic profile of caregivers (parents/guardians) of the children (n = 12).

Variables		Caregivers
Age (in years)	20-30 years	9 (75.0%)
	30-40 years	3 (25.0%)
Sex	Male	4 (33.3%)
	Female	8 (66.7%)
Education qualification	Illiterate	6 (50.0%)
	Primary	2 (16.7%)
	Middle school	1 (8.3%)
	High school	2 (16.7%)
	Higher secondary	1 (8.3%)
Occupation	Housewife	5 (41.7%)
	Elementary worker	6 (50.0%)
	Semiskilled worker	1 (8.3%)

Only two caregivers had ever heard of NRC prior to the admission of their children in the facility. However, all the children were admitted to the NRC for the first time. The majority of them were referred by doctors. All of them expressed being satisfied with the services provided in the facility (Table 3).

**Table 3:** Quantitative assessment of the experience of caregivers (parents/guardians) (n = 12) regarding the facility-based care of their children. NRC: nutrition rehabilitation centre; AWW: Anganwadi worker; ASHA: Accredited Social Health Activist.

Variables		Frequency
Presence of prior knowledge of the existence of the NRC		2 (16.7%)
First time visiting the NRC		12 (100.0%)
Referral to the NRC was done by	Doctor	10 (83.3%)
	AWW	1 (8.3%)
	ASHA	1 (8.3%)
Satisfaction with services in the NRC		12 (100.0%)

Service availability and readiness assessment of the facility

Presently, the facility is functioning with a five-member staff collaborating with the paediatrics department of the college for medical consultation. The two posts of attendant/helper and the post of medical social worker were found to be vacant. All the members of NRC had received training on SAM management, as depicted in Table 1. Documents essential for the management of SAM, ward equipment, kitchen and pharmacy supplies, and infrastructure were found to be available in the facility. Among the essential ward equipment, resuscitation and suction equipment were not available at the time of observation, as shown in Table 4.

**Table 4:** Details of service availability and readiness assessment of the facility in the management of SAM children. SAM: severe acute malnutrition; NRC: nutrition rehabilitation centre; SOP: standard operating procedure; IEC: information education communication; ORS: oral rehydration solution.

	Variables		Availability
1.	Number of beds		10
2.	Location		Within the medical college campus
3.	Human resources	Doctor (1)	1 (collaborated with the paediatrics department)
		Nurse (4)	3
		Nutritionist-cum-counsellor (1)	1
		Cook (1)	1
		Helper/attendant (2)	0
		Medical social worker (1)	0
4.	Documents related to SAM	Growth charts	Present
		SOPs	Present
		Operational guidelines of the NRC	Present
		IEC materials/wall charts	Present
5.	Essential ward equipment	Glucometer	Present
		Thermometer	Present
		Weighing scale	Present
		Infantometer	Present
		Stadiometer	Present
		Resuscitation equipment	Absent
		Suction equipment	Absent
6.	Other ward equipment	IV fluid stands	Present
		Almirahs	Present
		Room heaters	Present
		IEC - audio-visual materials	Present
		Toys for structural play	A few
		Clock	Present
		Calculator	Absent
		Reference height and weight chart	Present
7.	Kitchen supply	Cooking gas	Present
		Dietary scales (to weigh up to 5 g)	Present
		Electric blender (or manual whisks)	Present
		Water filter	Present
		Refrigerator	Present
		Utensils	Present
		Supply for making the starter and catch-up diet	Present
		Dried skimmed milk & whole dried milk	Absent
		Fresh whole milk	Present
		Puffed rice	Present
		Vegetable oil	Present
8.	Pharmacy supplies	Antibiotics	Present
		ORS	Present
		Multivitamin	Present
		Iron syrup	Present
		Folic acid	Present
		Vitamin A syrup	Present
		Zinc tablets	Present
		IV fluids	Present
		Cannula, IV sets, paediatric nasogastric tubes	Present
		Potassium chloride, magnesium chloride	Present
9.	Infrastructure and equipment	Patient register & counselling sheets	Present
		Dietary history sheet	Present
		Monthly reporting format	Present
		Linen & mattress	Present
		Washing & cleaning agents	Present
		Condition of the toilet	Acceptable
		Source of drinking water	Reverse osmosis (RO) purified
		Uninterrupted power supply	Present
		Fan & light	Present
		Sitting arrangement for the attendant	Present
		Sleeping arrangement for the attendant	Absent

Perspectives of the stakeholders on facility-based management of SAM

Opportunities and Strength

The majority of the key informants had highlighted several points indicating commitment and support at the level of government, administration, and community, which had helped the staff in the smooth conduction of their duties. Several caregivers expressed their satisfaction with the staff’s behaviour and dedication to delivering their services. Table 5 shows the details about the positive perspectives of the stakeholders.

**Table 5:** Details of the positive perspectives of key informants and caregivers (parents/guardians) on facility-based management of SAM. RBSK: Rashtriya Bal Swasthya Karyakram; DHS: Directorate of Health Service; ASHA: Accredited Social Health Activist; AWW: Anganwadi worker; NRC: nutrition rehabilitation centre; SAM: severe acute malnutrition; KI: key informant; CG: caregiver; C: code.

S. No.	Verbatim/statements	Descriptive code	Category	Theme
1	“We get the RBSK team from DHS while doing camps, people are happy if they get a doctor.” (KI-1) “Head of the Department of Paediatrics sends letters asking for referral from periphery.” (KI-1)	Health Department support (C-1)	Category 1. Government and community support	Opportunities
2	“If they (AWWs) find a patient they bring them to the facility.” (KI-1) “Follow up of the discharged cases is done by the ASHA and AWW in the community.” (KI-2)	Community level worker support (C-2)		
3	“We have good acceptance from the patient party.” (KI -5)	Patient party acceptance (C-3)		
4	“The food supply is sufficient. We give indent in a gap of 15-20 days and we get the supply as needed.” (KI-6)	Administrative commitment (C-4)		
5	“Our NRC is 24x7 full running well equipped facility.” (KI-2) “Whatever facility we have in NRC are good enough.” (KI-3)	Equipped facility (C-5)	Category 2. Self-sufficiency in service delivery	Strength of the facility
6	“The nursing staff is adequate.” (KI-5)	Adequate health personnel (C-6)		
7	“They give good food and in timely manner.” (CG-2) “The staff is good with us and teaches us how to feed our boy.” (CG-1) “The staff’s behaviour is good.” (CG-7)	Good communication (C-7)	Category 3. Dedication of staff	

Barriers and Challenges

The major challenge from the facility side was found to be a shortage of manpower and work overload among the staff. Almost all the key informants had expressed the need for at least one helper/attendant in the staff. The need for a security guard was also expressed by the key informants. Lack of emergency resuscitation equipment and oxygen cylinder was also felt by other key informants. Challenges from the beneficiary side were found to be responsibilities at home, poor knowledge of nutrition, out-of-pocket expenditure, and wage loss, as shown in Table 6.

**Table 6:** Summary of the perspectives of key informants and caregivers (parents/guardians), highlighting the barriers and challenges in facility-based management of SAM. SAM: severe acute malnutrition; PICU: paediatric intensive care unit; MCH: maternal and child health; KI: key informant; CG: caregiver; C: code.

S. No.	Verbatim/statements	Descriptive code	Category	Theme
1	“We need emergency resuscitation equipment in NRC.” (KI-2) “We do not have oxygen cylinder here.” (KI-3) “Because of lack of oxygen cylinder, we have to send the children to PICU in case of any emergency.” (KI-4) “We do not have facilities like oxygen cylinder for emergency; we have to shift the children to PICU.” (KI-5)	Emergency equipment shortage (C-8)	Category 4: Shortage in resources	Facility side barriers
2	“We do not have ward girl or any Grade IV employee or security guard. A few days back I had to take a child to PICU at 12.15 am. I was very afraid at that time of night.” (KI-4) “We need a care taker in the facility, in order to help the patient party to make receipts or to take them to do investigations or to take them to PICU, etc.” (KI-5) "We also need a security guard." (KI-5) “If there was a caretaker, it would have helped the people especially when giving directions to laboratories and MCH building. It would save our time.” (KI- 3)	Shortage of supporting staff (C-9)		
3	“We have deficiency of proper signage. So, we have to show them (caregivers) the way….. in between our own duties are delayed.” (KI-3) “We do our night duty alone. We have to shift them (children) to PICU all by ourselves in case of any emergency.” (KI-4) “We do not have a caretaker in the facility. I have to stay for the whole day. Apart from doing my own duties, I have to go and make receipts for the patients, take patients to the ward.” (KI-6)	Overburden of work (C-10)		
4	“Since most of the patients come with complications, the catch-up growth needs time…. the patients party finds it problematic to stay once the signs of co-morbidities subside.” (KI-2)	Presence of co-morbidities (C-11)	Category 5: Long duration of stay	Beneficiary side barrier
4	“I have another child at home. Who will take care of her if we stay here too long?” (CG-7). “We have elderly people at home; it was good if they let us go early.” (CG-5)	Responsibilities at home (C-12)		
5	“They (tea garden community) do not want to go outside and understand the outside society, do not eat adequate diet and even if resources are there, they do not utilize them.” (KI-1)	Hesitancy to accept outside help (C-13)	Category 6: Lack of awareness in the community	
6	“The guardians lack knowledge and awareness and the child is mostly neglected.” (KI-2) “Some of them (caregivers) do not know anything about nutrition… It takes a lot of our time.” (KI-3)	Poor knowledge of nutrition (C-14)		
7	“In some hard-to-reach areas, even 102 and 108 services are not available. They have to spend Rs 400-500 for transportation.” (KI-1)	Transportation problem (C-15)	Category 7: Financial loss	
8	“We rented a car to come from home; it costed Rs 2000.” (CG-1) “Till now we have spent Rs. 2000-3000. We have to buy this or that.” (CG-2). “ We have spent Rs 7000 till date, but it will be reimbursed by tea garden.” (CG-10) “Daily cost is around Rs 200-300.” (CG-6)	Out-of-pocket expenditure (C-16)		
9	“We are here for 29 days. I am a daily wage earner and I am unable to go to work.” (CG-9)	Wage loss (C-17)		
10	“We have low referral from periphery level health facilities.” (KI-1)	Low referral from peripheral health facility (C-18)	Category 8: Lack of commitment from the peripheral health facility	Community side barrier

## Discussion

The NRC in Jorhat was established in April 2015. The Government of India has laid down operational guidelines for the smooth functioning of the NRCs. This document clearly points out various aspects, such as human resources, infrastructure, and training for capacity building, along with protocols for the management of malnutrition and associated co-morbidities. However, irrespective of this, any facility established must face its own set of challenges and avail its own set of opportunities while imparting services to the beneficiaries.

The present study aims to assess the readiness of the facility to deliver services and to highlight the various challenges faced by the stakeholders of the NRC. While assessing the service availability and readiness of the facility, we observed the lack of required manpower in the NRC staff. The deficiency of a designated medical officer has been compensated for by collaborating with the medical college. However, the deficiency of helper/attendant and medical social worker (MSW) remains unfulfilled. Moreover, the facility lacked resuscitation and suction equipment, which are vital for managing any emergency. Also, no separate sleeping arrangements were present for the caregivers. Other than these, the facility was found to be functioning with sufficient other ward equipment and kitchen and pharmacy supplies.

Our findings can be compared with the study done by Tandon et al. [13], which also reported a vacancy of essential staff. In this study, they found 68% of the cases to be first-time visitors to the NRC. However, in our study, all the caregivers informed us that they had brought their children to the NRC for the first time.

Another study done by Fahim et al. [14] reported a similar deficiency of sleeping facility, together with a sitting arrangement deficiency for the caregivers. This study also reported a deficiency of essential equipment, together with a deficiency of food supply, manpower, increased workload, insufficient space, reluctance of caregivers in completing the treatment regimen, insufficient knowledge of caregivers regarding feeding practices, an excess number of attendants with cases, and poverty of the parents.

All the key informants had pointed out several important opportunities while carrying out the functioning of the facility. The support of the Directorate of Health Services through Rashtriya Bal Swasthya Karyakram (RBSK), continuous support from the medical college administration to meet the demand and supply of logistics, and support of the department of paediatrics in the management of SAM children were some of the positive perspectives of the key informants.

In our study, several caregivers had pointed out the timely availability of food, supportive behaviour of the staff, nutrition counselling, etc., highlighting the commitment of the staff to their respective roles.

The barriers faced by various stakeholders were divided into three themes: facility-side barriers, beneficiary-side barriers, and community-side barriers.

The major facility-side barriers were “the need of emergency resuscitation equipment in NRC” and “lack of oxygen cylinders”. Almost all the key informants had pointed out the shortage of supportive staff in the facility: “We do not have ward girl or Grade IV staff”, “We need a caretaker in the facility”, “We also need a security guard,” etc.

Shortage of the above-mentioned human resources has led to the overburden of work among the existing staff, for example, “Have to shift children to PICU all by ourselves in case of emergency” and “Have to show caregivers the directions to laboratories, wards, etc., interrupting our own respective duties”. The availability of proper signage to the necessary facilities and the presence of a helper/attendant could have reduced the workload of the existing staff. Moreover, the availability of a designated medical officer for NRC and the missing emergency equipment could have helped in the management of emergencies in the facility itself.

Challenges from the beneficiary side were found to be responsibilities at home (“I have another child at home”, “We have elderly people at home”), poor knowledge of nutrition, out-of-pocket expenditure, and wage loss leading to financial troubles. Both key informants and caregivers had reported the long duration of stay in the facility as a challenge. The key informants reported a lack of motivation on the beneficiary side to stay for such a long duration. The key informants also highlighted that “once the symptoms of comorbidities subside, the caregivers want to take their child back to home”. Constant counselling would be required thereafter to retain the cases in the NRC (KI-5). These prevent proper growth catch-up in cases, which generally begins after proper management of associated co-morbidities. The reasons for reluctance of the caregivers to stay for the required duration were mainly unattended responsibilities at home.

The key informant, KI-2, suggested, “If we can involve the CHCs and PHCs in facility based management of SAM; then after initial management in NRC the cases could have been referred to such facilities near the residence of the cases. In this manner, the compliance of the beneficiaries could have been increased”. Creating a referral linkage with various health facilities within a district can be taken into consideration by policymakers, which might help in increasing compliance.

The main barrier at the community level was poor referral of SAM cases from peripheral health facilities: “We get most of the SAM cases from our own medical college OPD and Indoor wards and the health camps that we organise in the community” (KI 1). The key informant also added, “Initiatives have been taken by HOD paediatrics to send letter asking for referral from peripheral health facilities” (KI-1).

Limitations of the study

As our study was a facility-based study, key community-level stakeholders like ASHA and Anganwadi workers could not be included in the study. Moreover, our study was conducted purposively in a single accessible NRC; further multicentric studies in the future might provide us with more generalizable findings.

## Conclusions

The present study has highlighted various challenges, both from the healthcare provider and beneficiary side, involving diverse areas such as human resources, financial issues, emergency supplies, workload, nutritional awareness, and time constraints. However, all the beneficiaries expressed satisfaction with the services in the NRC.

## Appendices

Key informant interview guide

Name of interviewer 1 ______________________________

Name of interviewer 2 ______________________________ Date _____________

Background script

Greetings,

My name is _____________________. I am working as ___________. Thank you for joining us today. We are doing a project on assessing the opportunities and barriers in facility-based management of Severe Acute Malnutrition among children in Jorhat, Assam. As a part of the project, we are interviewing the stakeholders who are involved in facility-based management of SAM children at the facility level. We would like your valuable feedback and insight on the opportunities available and barriers faced by you during the management of SAM children at the facility level. Your openness and honesty on the topic will be highly appreciated.

Information learned from this interview will help us better understand the functioning of the facility and the challenges faced.

Anonymity regarding the identity of the stakeholders will be maintained.

We would like your permission to record the interview. We would also like to seek your permission to use the quotes from the interview.

The interview will take 15 to 20 minutes.

If you have any queries, please feel free to ask.

Is it OK to begin the interview?

1. Sex of the interviewee___________

2. What is your age?

3. What is your educational qualification?

4. What is your current designation?

5. What is your affiliation?

6. How long have you been associated with facility-based management of SAM children?

7. How long have you been working in this facility?

8. Have you received training on facility-based management of SAM?

9. What is your view on the current status of functioning of facility-based management of SAM children in Jorhat?

10. What initiatives/opportunities/actions are you aware of that address the capacity building of the workforce?

11. What are the gaps/barriers you feel that are hampering the smooth functioning of facility-based management of SAM?

12. What are the initiatives/actions taken to address these barriers?

13. What is your opinion on these actions taken? Are they sufficient?

14. What suggestions do you want to give to address the barriers and improve the functioning of facility-based management of SAM?

Conclusion:

Thank you for your time and valuable information.

In-depth interview guide for caregivers

Name of interviewer 1 ______________________________

Name of interviewer 2 ______________________________ Date _____________

Background script

Greetings,

My name is _____________________. I am working as ___________. Thank you for joining us today. We are doing a project on assessing the opportunities and barriers in facility-based management of Severe Acute Malnutrition among children in Assam. As a part of the project, we are interviewing the caregivers of the SAM children admitted to the NRC. We would like your valuable feedback and insight on the opportunities available and barriers faced by you and your child during facility-based management. Your openness and honesty on the topic will be highly appreciated.

Information learned from this interview will help to better understand the functioning of the facility and the challenges faced.

Anonymity regarding the identity of the stakeholders will be maintained.

We would like your permission to record the interview. We would also like to seek your permission to use the quotes from the interview.

The interview will take 15 to 20 minutes.

If you have any queries, please feel free to ask.

Is it OK to begin the interview?

1. Age of the caregiver - ______________years

2. Sex of the caregiver - M/F

3. Education qualification - Illiterate/below primary/primary/middle school/high school/HS/diploma/graduate/postgraduate

4. Occupation - Unemployed/Housewife/Elementary occupation/Agriculture/Service/Business_____________

5. Did you know about the existence of a Nutrition Rehabilitation Centre for children with malnutrition? Yes/No, if yes, who told you__________________

6. Is this your first time visiting an NRC? Yes/No

7. If no, then specify the number of visits done before_____________

8. Who referred the child to the NRC? AWW/ANM/Physician/Others____________

9. Are you satisfied with the services? Yes/No

10. According to you, what are the positive points in facility-based management of SAM children?

11. What are the barriers/challenges that you faced during the treatment of your child under the programme?

a) Patient care and treatment

b) Facilities for attendants, like food and stay

c) 24-hour water supply

d) Food

e) Availability of 24-hour electricity

f) Counselling of the caregivers on care and hygiene

g) Transportation to the facility

h) Out-of-pocket expenditure

i) Others___________________________________________________________________________________________________________

12. What suggestions do you want to give to remove or minimise those barriers?

Conclusion:

Thank you for your time and valuable information.

Observation guide

1. Number of beds in NRC - 10/20

2. Availability of required manpower

Doctor _________ Nurse_________

Nutritionist cum counsellor_______ Cook_________

Helper/Cleaner___________ Medical Social Worker_________

3. Availability of required documents related to SAM

Growth charts_______

SOPs________

Operational guidelines of NRC________

IEC materials/Wall charts_________

4. Availability of essential ward equipment required for NRC

Glucometer____

Thermometer_____

Weighing scale_______

Infantometer_____

Stadiometer______

Resuscitation equipment_____

Suction equipment______

5. Availability of other ward equipment

IV stands

Almirahs

Room heaters

IEC - Audio/visual material (TV/DVD)

Toys for structural play (Safe, homemade)

Clock

Calculator

Reference height and weight chart

6. Availability of kitchen supplies

Cooking Gas

Dietary scales (to weigh up to 5 g)

Measuring jars, electric blender (or manual whisks)

Water filter

Refrigerator

Utensils (large containers, cooking utensils, feeding cups, saucers, spoons, jugs, etc.)

Supply for making the starter and catch-up diet

Dried skimmed milk

Whole dried milk

Fresh whole milk

Puffed rice

Vegetable oil

Foods similar to those used in the home

7. Availability of pharmacy supplies

Antibiotics

ORS

Electrolytes and minerals

Potassium chloride

Magnesium chloride/sulphate

Iron syrup

Multivitamin

Folic acid

Vitamin A syrup

Zinc sulfate or dispersible zinc tablets, glucose (or sucrose)

IV fluid

Cannulas, IV sets, paediatric nasogastric tubes

8. Infrastructure and equipment required at NRC

Office furniture

Patient register

Counselling sheets

Monthly report of children admitted to the NRC

Dietary history sheets

Monthly reporting format

Linen

Mattress

Washing & cleaning agents: phenyl, acid, soap, and detergent

Condition toilet

Source of drinking water

Uninterrupted electricity supply

Fan

Light

Sitting arrangement for the attendant

Sleeping arrangement for the attendant
